# Case Report: Evidence-based case management of ECMO treatment for children with severe lung abscess complicated with bronchopleural fistula

**DOI:** 10.3389/fped.2025.1676151

**Published:** 2025-11-10

**Authors:** Li Dan, Meng Yuqian

**Affiliations:** Department of PICU Children’s Hospital, Chongqing Medical University, Chongqing, China

**Keywords:** necrotizing pneumonia, extracorporeal membrane oxygenation, bronchialoccluder, prone position ventilation, pressure injury, evidence-based nursing

## Abstract

This report details an evidence-based nursing management protocol for pediatric cases of severe lung abscess complicated by bronchopleural fistula treated with extracorporeal membrane oxygenation (ECMO). Evidence-based targeted nursing interventions were implemented, including: prone position ventilation care, closed-chest drainage care, bronchial blocker monitoring and care, ECMO cannulas management and hemorrhage control, and innovative measures to prevent pressure injuries related to tubing and devices, The patient underwent 16 days of ECMO support, spent 58 days in the ICU, and was discharged after a total hospital stay of 70 days with full recovery. Follow-up at 3 months post-discharge confirmed excellent pulmonary function restoration.

## 1 Background

Necrotizing pneumonia (NP) represents the most severe form of pediatric lung abscess and is a serious complication of severe pneumonia. It is characterized by pulmonary consolidation with liquefactive necrosis, ultimately leading to cavity formation within the lung parenchyma (1, 2). In recent years, the incidence of NP in children has shown a rising trend. Clinically, NP most commonly presents with fever and cough, and is frequently associated with severe illness, rapid progression, and multiple complications. Notably, approximately 17%–67% of patients with NP develop bronchopleural fistula (BPF), which is strongly associated with increased morbidity and poor outcomes (3). In critical cases, NP can further progress to septic shock and multi-organ dysfunction. Extracorporeal membrane oxygenation (ECMO) has emerged as a life-saving therapeutic option for patients with end-stage respiratory failure, providing essential cardiopulmonary support when conventional management is insufficient (4). Herein, we present a nursing case report of a pediatric patient with severe NP complicated by BPF, who was admitted to our ward in June 2024. The patient was successfully discharged after 16 days of ECMO support, 58 days in the ICU, and a total hospital stay of 70 days. An evidence-based summary is provided in this report, which was approved by the hospital ethics committee (Approval No. 2024-Ethics-Clinical-526).

## 2 Case presentation

A 7-year-old girl was admitted to the hospital on June 12 with a history of cough lasting more than 20 days and intermittent fever for 20 days. On June 19, due to clinical deterioration, she was transferred from the Respiratory Ward to the Pediatric Intensive Care Unit (PICU). At admission to the PICU, her vital signs were as follows: body temperature 39°C, heart rate 178 beats/min, respiratory rate 55 breaths/min, oxygen saturation 88%, and blood pressure 90/64 mmHg. She exhibited severe respiratory distress with dyspnea, cyanosis of the lips, and evident suprasternal and intercostal retractions. Endotracheal intubation was performed immediately for ventilatory support. Physical examination revealed slightly cool extremities with capillary refill time of 2.5–3 s. Cardiac auscultation detected strong heart sounds with a regular rhythm, without murmurs at either the apex or base. Neurological assessment showed bilaterally equal pupils with sluggish light reflexes, and her Glasgow Coma Scale (GCS) score was 8.

**Figure 1:**
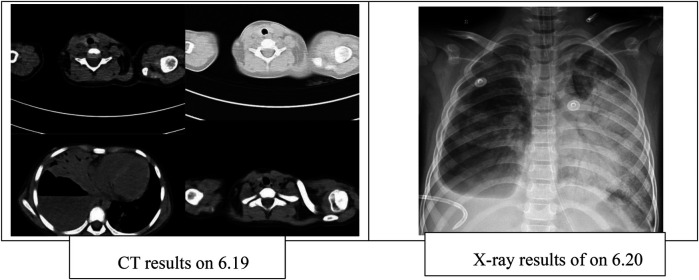
Pulmonary condition.

Chest CT performed on June 19 revealed a large cavitary lesion in the lower lobe of the right lung measuring approximately 86.7 × 84.3 mm, accompanied by right-sided pleural effusion. A chest x-ray on June 20 demonstrated increased lung lucency bilaterally (Figure 1). Despite mechanical ventilation with 100% FiO_2_, her oxygen saturation remained at 78%, and arterial blood gas analysis showed a PaO_2_ of 36.4 mmHg. Therefore, veno-arterial extracorporeal membrane oxygenation (VA-ECMO) was initiated. During ECMO support, right-sided closed thoracic drainage was performed, and the right lung was isolated with a bronchial blocker. The patient also received broad-spectrum antimicrobial therapy, analgesia and sedation, skin care, nutritional support, and other comprehensive management. After 16 days of ECMO therapy, she was successfully weaned and transitioned to high-frequency oscillatory ventilation. On July 11, she was further switched to conventional mechanical ventilation, and on July 28, the microwave therapy device [BXING, WB-3100 (AIII)] delivers localized irradiation to the patient's lungs in a pulsed mode (operating frequency: 2450 MHz, power: 60 W, vertical radiation from the microwave probe at a distance of 5 cm from the skin surface). Treatment was administered 20 min before and after tracheal tube removal. This therapy is primarily used to improve local blood circulation, alleviate pain, and promote the resolution of inflammation. microwave therapy (5). Following extubation, the patient received 10 min of face mask nebulization therapy, immediately transitioning to nasal cannula oxygen therapy. On August 16, she was transferred back to the Respiratory Ward and was discharged on August 21, after a total hospital stay of 70 days.

## 3 Management and outcome

### 3.1 Evidence-based respiratory management

Based on the patient's clinical condition and the 2020 Expert Consensus on Airway Clearance Technology (6), the following respiratory measures and ventilation strategies were implemented to improve oxygenation and promote lung recovery. The primary objectives were to facilitate alveolar re-expansion and enhance oxygenation.

#### 3.1.1 Prone ventilation

Studies have shown that prone positioning ventilation significantly improves the oxygenation index in pediatric patients with ARDS (7, 8).Recent evidence recommends 12–16 h of prone positioning ventilation per day (9). During ECMO support, a standardized prone positioning protocol was followed in accordance with the “Safety and Comfort Care Protocol for Prone Positioning Ventilation Patients” (10) and the “Adult Prone Positioning Ventilation Group Standard” (11). Turning was performed by a team of 3–5 staff members positioned at the patient's head, neck, shoulders, and hips. The patient was repositioned every 2 h from the side with more tubing to the side with less tubing, using a 30° turning support pad.During prone ventilation, careful monitoring of pressure-prone skin areas is essential. Studies have demonstrated that this technique can increase the PaO_2_/FiO_2_ ratio by approximately 50–70 mmHg (12).Arterial blood gases were monitored every 4 h to assess the effectiveness of prone ventilation (13). In a case reported by Wang Jun et al., oxygenation significantly improved, supporting the efficacy of prone ventilation in pediatric ARDS (14).

#### 3.1.2 Bronchopulmonary lavage

Bronchoscopy is indicated following pulmonary consolidation.Due to the severity of the condition, repeated bronchoscopic procedures are often required (15). Upon admission, the patient underwent multiple fiberoptic bronchoscopies with alveolar lavage. As oral intubation was required, a three-way connector and a dental pad were prepared in advance. Throughout the procedure, the patient's vital signs were closely monitored, and the lavage was performed using a 2% acetylcysteine solution under medical supervision. Each lavage used 5–10 mL/kg of solution, and vital signs were recorded before and after the procedure, under strict aseptic conditions. A recent meta-analysis indicates that bronchoalveolar lavage significantly reduces the duration of mechanical ventilation in severe pneumonia (mean difference: −2.3 days; 95% CI: −3.1 to −1.5) (16). Repeated bronchoscopic treatments assist in clearing necrotic tissue and promoting lung re-expansion (13).

#### 3.1.3 Closed thoracic drainage with bronchial blocker

Given the presence of a large cavitary lesion in the right lung accompanied by pleural effusion, closed-chest drainage was initiated. Bubbles and fluid were continuously evacuated, with the volume and characteristics of the drainage fluid carefully monitored. The right lung abscess was associated with severe air leakage. To address this, a 7-Fr bronchial blocker was inserted into the middle segment of the right lung under bronchoscopic guidance, enabling selective single-lung ventilation and preventing inflammatory secretions and air from entering the left lung. Nursing staff conducted strict monitoring of the blocker during each shift, measuring the exposed length of the cannulas to detect displacement, inspecting for bubbles in the drainage bottle, and adjusting the balloon inflation volume based on bubble levels. Previous studies have demonstrated that bronchial blockers are effective in controlling air leakage in patients with bronchopleural fistula (BPF) and contribute to improved oxygenation (17). This finding aligns with evidence supporting continuous closed drainage strategies for BPF management (14). In collaboration with physicians, nurses adjusted the blocker's position when necessary, and the device remained in place until hospital day 41. Closed-chest drainage was continued until hospital day 56, at which point the drainage tube was removed without any evidence of secondary infection.

### 3.2 Precise management during ECMO

#### 3.2.1 Anticoagulation and bleeding balance management

Bleeding during ECMO is often associated with coagulation abnormalities caused by systemic heparinization. Monitoring coagulation status and implementing individualized anticoagulation therapy guided by activated clotting time and thromboelastography is recommended (18). This patient presented with bleeding at the cannulas insertion site and oronasal hemorrhage. Initial management involved nasal packing with vaseline gauze and prompt removal of oral blood clots. Subsequently, the insertion site was packed with isoproterenol-soaked gauze, followed by application of thrombin gel and external bandaging to achieve hemostasis.

#### 3.2.2 Management of cannulas and oxygenators

Latex pads were used under ECMO cannulas to reduce local pressure and prevent device-related pressure injuries. Previous studies have emphasized the importance of regularly assessing of oxygenator function (19). In this case, 14-Fr and 19-Fr heparin-coated cannulas sheaths (Bio-Medicus; Medtronic, Minneapolis, MN, USA) were surgically implanted into the right common carotid artery and internal jugular vein, respectively. The oxygenator was the Hilite 2400LT (Xenios AG, Germany).Pre-treatment ECMO pump parameters were set as follows: rotor speed(5,000 rpm); blood flow(1.2 L/min); gas flow(1.2 L/min); and FiO_2_(100%). The oxygenator was not replaced during treatment.

#### 3.2.3 Skin management

**Figure 2:**
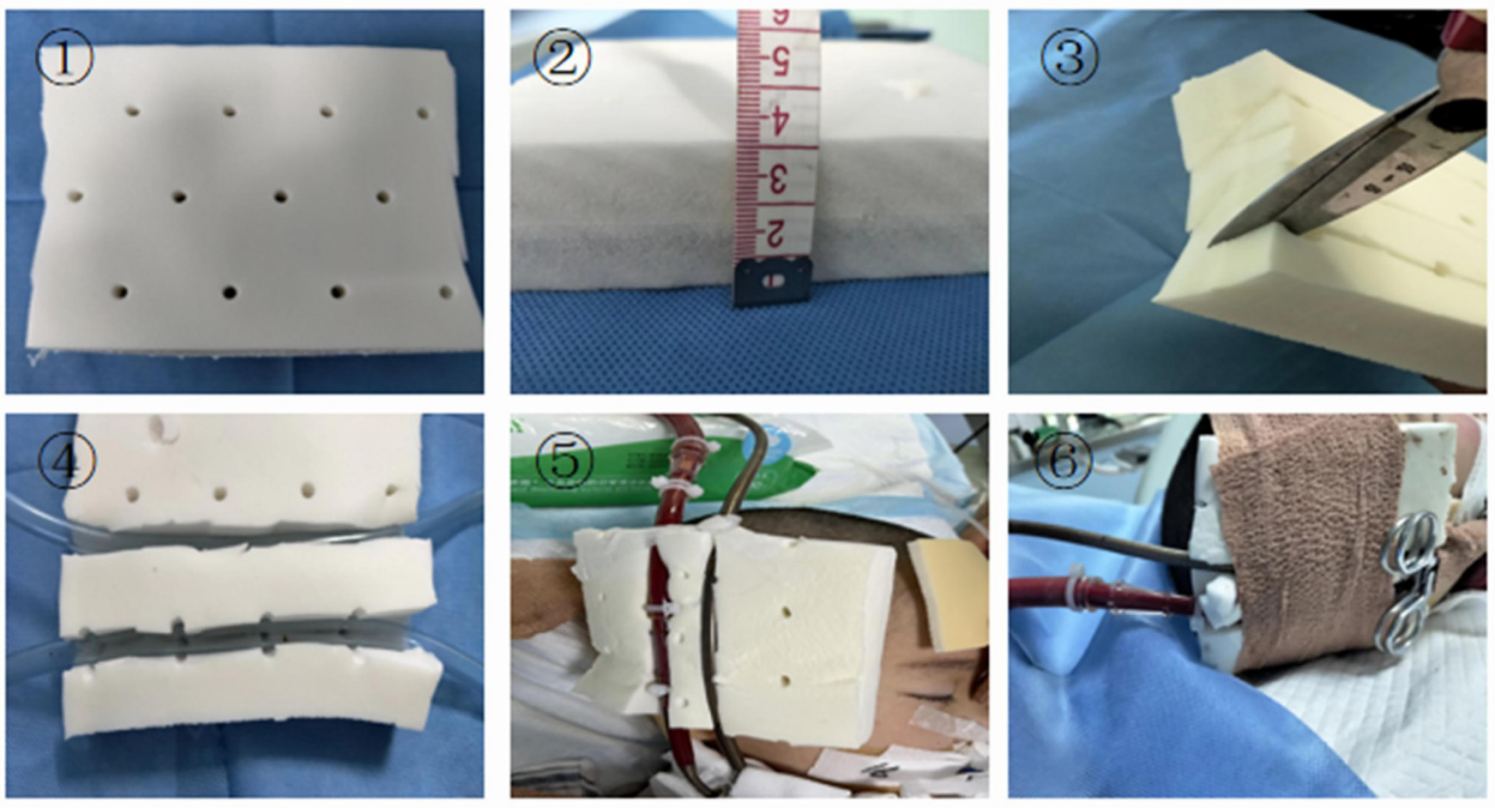
Improved latex pad with elastic bandage. (1) Latex pad. (2) The latex pad is 3 cm thick. (3) Cut the latex pad from the middle, taking care not to cut all the way through. (4) Latex pad after cutting. (5) Embed the ECMO tubing into the latex pad. (6) Secure the latex pad and catheter with an elastic bandage.

The BRAND-Q scale assessment indicated a high risk of pressure injury. The patient's ECMO arterial and venous cannulae required reinforced fixation to prevent displacement, yet their large diameter and rigid texture made insertion sites highly susceptible to pressure ulcers. Studies have shown that customized pressure-relief devices can reduce medical device–related pressure ulcers by approximately 65% (18). Accordingly, we innovatively employed latex pads with a 2 cm slot cut to accommodate dual cannulae, achieving effective pressure relief through skin fixation (Figure 2). In addition, prolonged prone positioning further increased the risk of pressure injury. To address this, air-filled mattresses, water pillows, and silicone foam dressings were applied to vulnerable sites. With these protective interventions, no skin injuries occurred during the patient's hospitalization.

## 4 Discussion

In this case, prone ventilation, bronchoalveolar lavage, a bronchial blocker, and ECMO were used to successfully manage a child with complex necrotizing pneumonia complicated by BPF. Evidence-based guidelines recommend prone ventilation for at least 12 h per day in patients with severe ARDS to improve oxygenation and reduce mortality (20, 21). In this case, prone ventilation was applied for 12 to 16 h per day, consistent with the guidelines. It also facilitated re-expansion of the affected lung segments, improved ventilation-perfusion matching, and minimized further injury to the healthy lung. Prone ventilation during ECMO is technically more challenging, particularly in the presence of significant bleeding, as observed in this child. Each repositioning required a team of five staff members, and the envelope method was employed to ensure safety. One person is specifically responsible for ensuring the safety of the ECMO cannulas during prone positioning, and the innovative use of slotted latex pads for decompression effectively prevented pressure injuries.

Bronchoalveolar lavage is important for removing mucus plugs and purulent secretions, as well as for obtaining diagnostic samples to guide antibiotic therapy (22, 23). However, when large amounts of pus are continuously drained through the trachea, or when pus flows into the contralateral lung, repeated lavage alone may not fully eliminate the source and may temporarily worsen oxygenation and ventilation stability. Therefore, if lavage is ineffective and contamination persists, additional strategies such as local isolation or surgical intervention should be considered. In this case, a 7Fr bronchial blocker was inserted into the right middle lobe via bronchoscopy to achieve selective single-lung ventilation. This technique is relatively simple and reversible, and is suitable as a short- to medium-term bridge when persistent unilateral pus or bleeding contaminates the contralateral lung and immediate surgical resection is not feasible. After placement, close monitoring is required for potential effects on oxygenation and ventilation, secondary infection, displacement of the blocker, and changes in bubbling within the drainage tube. If bubbling persists after seven days of observation, re-exploration with repositioning or alternative treatments should be considered. This approach merits broader clinical application.

## 5 Conclusion

This report demonstrates that comprehensive, evidence-based nursing management plays a pivotal role in the successful treatment of pediatric necrotizing pneumonia complicated by bronchopleural fistula supported with ECMO. The integration of prone positioning ventilation, bronchial occlusion, bronchoalveolar lavage, and individualized ECMO nursing strategies—including meticulous cannula management, and innovative pressure injury prevention—contributed to the restoration of pulmonary function and complete clinical recovery. These findings underscore the importance of systematic, evidence-guided nursing interventions in optimizing respiratory support, minimizing complications, and enhancing patient outcomes in complex pediatric ECMO cases. Continued refinement and standardization of such nursing protocols are essential to further improve survival rates and long-term prognoses in critically ill children requiring extracorporeal life support.

## Data Availability

The original contributions presented in the study are included in the article/Supplementary Material, further inquiries can be directed to the corresponding author.
